# Depressive symptoms are associated with fatigue, poorer functional status and less engagement in sports in axSpA and PsA: an analysis from the RABBIT-SpA cohort

**DOI:** 10.1186/s13075-023-03127-2

**Published:** 2023-08-02

**Authors:** Andreas Reich, Anja Weiß, Lisa Lindner, Xenofon Baraliakos, Denis Poddubnyy, Silke Zinke, Carsten Stille, Anja Strangfeld, Anne C. Regierer

**Affiliations:** 1 German Rheumatism Research Center (DRFZ Berlin), Epidemiology and Health Services Research, Berlin, Germany; 2 Ruhr University Bochum, Bochum, Germany; 3 Rheumazentrum Ruhrgebiet, Herne, Germany; 4 Charité – Universitätsmedizin Berlin, Department of Gastroenterology, Infectiology and Rheumatology, Berlin, Germany; 5 Berlin, Germany; 6 Hannover, Germany; 7 Charité – Universitätsmedizin Berlin, Berlin, Germany

**Keywords:** Observational study, Spondyloarthritis, Psoriatic arthritis, Depression

## Abstract

**Background:**

In patients with axial spondyloarthritis (axSpA) or psoriatic arthritis (PsA), concomitant depression might have a negative impact on the course of disease and treatment outcomes. The aims of this analysis are to determine the prevalence of depressive symptoms in axSpA and PsA patients in a real-world cohort study and to identify sociodemographic and clinical associated factors for moderate or severe depressive symptoms in both diseases.

**Methods:**

Patients from the RABBIT-SpA cohort with an axSpA or PsA diagnosis and a valid WHO-5 Well-Being Index score at baseline were included. A descriptive analysis of baseline and outcome parameters by category of depressive symptoms was performed and factors associated with the presence of depressive symptoms (moderate or severe) were examined in a logistic regression.

**Results:**

Two thousand four hundred seventy patients (1,245 axSpA; 1,225 PsA) were included in the analysis. In both diagnoses, the proportion of patients with moderate depressive symptoms was 8% and 21% with severe symptoms.

Patients with moderate or severe depressive symptoms were less likely to engage in sports than those with no or mild depressive symptoms, had more comorbidities and higher scores for disease activity, functional limitations, fatigue, and pain and took more analgesics.

In axSpA, patients with a higher disease activity, a greater functional impairment and more severe fatigue were more likely to experience depressive symptoms, while patients with more years in education and engaging in sports for at least 1 h/week were less likely to experience depressive symptoms. PsA patients with a greater functional impairment and more severe fatigue were more likely to experience depressive symptoms while those engaging in sports for at least 1 h/week were less likely to experience depressive symptoms.

**Conclusion:**

We confirmed a high prevalence of depressive symptoms in both PsA and axSpA. Factors negatively associated with the presence of depressive symptoms were fatigue, not engaging in sports, and greater functional limitations. Depressive symptoms may affect the perception of disease activity / severity by patients. Thus, depressive symptoms are an important condition in axSpA and PsA that should be considered when evaluating disease activity and treatment outcomes.

**Supplementary Information:**

The online version contains supplementary material available at 10.1186/s13075-023-03127-2.

## Background

Axial spondyloarthritis (axSpA) and psoriatic arthritis (PsA) are chronic inflammatory diseases that result in a high burden of disease and reduced function and quality of life [1, 2]. AxSpA is characterised by chronic inflammatory back pain as its main symptom, often starting in the third decade of life. The most common peripheral manifestations are arthritis and enthesitis. Extra-articular manifestations can occur, such as acute anterior uveitis, psoriasis or inflammatory bowel disease [2]. PsA is a multifaceted disease that affects up to 30% of psoriasis patients [1]. Commonly, musculoskeletal symptoms start after years of psoriatic skin manifestations but, in approximately 15% of patients, both can appear at the same time or arthritis can start before any skin symptoms. Those affected suffer from painful inflammation of the joints or spine as well as pathological changes in the skin and nails.

Chronic pain and depression often occur together, causing a greater burden of disease than either condition alone; pain can lead to depression, and depression can increase the perception of physical pain [3]. In both axSpA and PsA, the relevant scores used to assess treatment success and to guide therapeutic decisions are either informed by patient reported outcome measures (PROMs), such as the Bath Ankylosing Spondylitis Disease Activity Index (BASDAI) or Bath Ankylosing Spondylitis Functional Index (BASFI) for axSpA or are composite scores containing PROMs (Ankylosing Spondylitis Disease Activity Score c-reactive protein (ASDAS-CRP) for axSpA and Disease Activity in Psoriatic Arthritis (DAPSA) for PsA). Thus, recognizing an existing depression in chronic pain patients and treating both conditions is of great importance.

A high prevalence of depressive symptoms has been shown for axSpA [4–6] and PsA patients, though for the latter, data is still comparatively rare [7, 8]. While data is still scarce, there are some studies suggesting that depressive symptoms independently, negatively influence treatment outcomes in rheumatoid arthritis [9], axSpA [10] and PsA [11].

The aims of this analysis are to 1) determine the prevalence of depressive symptoms in axSpA and PsA patients in the RABBIT-SpA cohort study, 2) identify sociodemographic and clinical differences between patients with no or mild depressive symptoms and those with moderate or severe symptoms, 3) analyse associated factors of depressive symptoms in axSpA and PsA patients.

## Methods

### Patients

Rabbit-SpA is an observational, longitudinal cohort study, started by the German Rheumatism Research Centre in 2017. The main aim of the study is to examine long-term safety and effectiveness of biologic and targeted synthetic disease-modifying antirheumatic drugs (b/tsDMARDs) in patients with a diagnosis of either axSpA or PsA. Participating rheumatology clinics or practices all over Germany enrol patients with at least one prior failure of a systemic therapy. Patients receiving a new b/tsDMARD are included in the treatment group, while patients receiving a conventional systemic treatment are included in the control group. Patients of both groups were included in this analysis.

Documentation is web-based, however, patients can also fill in paper questionnaires in the study centres. Both physicians and patients fill in case report forms (CRFs) at baseline, after 3 months and 6 months and every 6 months thereafter, for a maximum follow-up time of 10 years.

The physician CRF contains data on onset of first symptoms, first contact with a rheumatologist and date of first diagnosis. Information on comorbidities and extra-articular manifestations is collected as well as current and past antirheumatic therapy, both medical and non-medical. Disease activity is measured using a variety of instruments, including established composite scores of disease activity, clinical parameters such as laboratory values and imaging results. The patient questionnaire contains sociodemographic information and a number of PROMs (for example, BASDAI and BASFI for axSpA and the Dermatology Life Quality Index (DLQI) and Health Assessment Questionnaire (HAQ) for PsA). A more detailed description of the study can be found elsewhere [12, 13].

All patients recruited since the beginning of the study (May 2017) until database closure on March 1st, 2022 were included in the present analysis if they had a baseline physician’s CRF and a baseline patient’s CRF with a complete WHO-5 Well-Being Index (WHO-5).

### Definition of variables

#### Depressive symptoms

Depressive symptoms were measured with the 5-item WHO-5, which is frequently used as a screening tool for depression [14]. It consists of 5 questions assessing subjective psychological well-being in the last 14 days, on a scale of 0 (never) to 5 (all of the time):

- 1: ‘I have felt cheerful and in good spirits’, 2: ‘I have felt calm and relaxed’, 3: ‘I have felt active and vigorous’, 4: ‘I woke up feeling fresh and rested’ and 5: ‘My daily life has been filled with things that interest me’.

The scores of the single items are added and multiplied by 4 to obtain a WHO-5 percentage score between 0 and 100. Established cut-off values [5, 15–17] were used to separate the score into the following four groups: Severe depressive symptoms indicated by values < 13, values between 13 and 28 suggest moderate depressive symptoms, values between 29 and 50 mild depressive symptoms, while values above 50 indicate good well-being and no depressive symptoms.

#### Independent variables

Independent variables were selected a priori, based on findings from the literature [2, 18, 19]. Sex, disease duration (years since diagnosis), obesity (BMI >  = 30), arthritis (only in axSpA patients, 0–44 joints, dichotomized to yes (1 or more affected joints) vs. no), and enthesitis (0–16 affected enthesial points, dichotomized to yes (1 or more affected points) vs. no) were physician reported. Data on comorbidities was also collected from the rheumatologist, using a checklist of comorbidities relevant to axSpA and PsA, including for example cardiovascular diseases but also depression. The checked comorbidities were counted and the result categorized (‘0–2 comorbidities’ vs. ‘3 or more comorbidities’). Education (dichotomized to >  = 10y vs. < 10y) and engaging in sports (‘How often do you engage in sport?’ with possible answers ‘more than 4 h a week’, ‘2 to 4 h a week’,’1 to 2 h a week’, ‘less than one hour a week’ and ‘do not engage in sport’) were collected in the patient CRF. Disease activity was measured with the instruments recommended for the respective diagnosis (ASDAS (CRP) for axSpA, DAPSA for PsA) by an international task force [20]. Functional impairment was measured by BASFI in axSpA and HAQ in PsA. In axSpA patients, fatigue was measured with the first BASDAI question: ‘How would you describe the overall level of fatigue / tiredness you have experienced [in the last 7 days]?’ on a scale from 0 ‘no fatigue/tiredness’ to 10 ‘very severe fatigue/tiredness’. In PsA patients, the second question of the Psoriatic Arthritis Impact of Disease 12-item questionnaire was used: ‘How severe was your tiredness / fatigue due to your psoriatic arthritis in the last week?’ on a scale from 0 ‘no tiredness’ to 10 ‘complete exhaustion’. Also in PsA patients, body surface area (BSA, physician-reported, 0–100%) was used to measure the extent of skin involvement and DLQI (patient reported) to measure the impact of skin involvement on quality of life.

#### Statistical analysis

Except for c-reactive protein (CRP) and disease duration, the proportion of missing values for analysis parameters was between 0 and 3%. For CRP, there were 15% missing values in axSpA patients and 11% in PsA patients. The ASDAS (CRP) score used in axSpA patients also had 15% missing values. For disease duration it was 7% and 9%, respectively. Therefore, data were imputed multiply (10 times), using fitted regression models [21]. Combined estimates were obtained by combining imputations.

For both diagnoses descriptive statistics as means (± standard deviation) and percentages were used to compare patients with ‘severe depressive symptoms’, ‘moderate depressive symptoms’ and ‘no or mild depressive symptoms’.

To identify sociodemographic or clinical factors that are associated with depressive symptoms, moderate and severe depressive symptoms (WHO-5 ≤ 28) were combined and compared to patients with no or mild depressive symptoms. For these two groups, a multivariate logistic regression was used to determine associations between independent variables (sex, education, comorbidities, engaging in sports, disease activity, physical function, and fatigue) and depressive symptoms. Adjusted odds ratios (OR) are given with 95% confidence intervals (95% CI).

Separate regression models were fitted for axSpA and PsA.

Calculations were carried out with the software package SAS, version 9.4 (SAS Institute, Cary, USA) and R, version 3.5.3 (R Foundation for Statistical Computing, Vienna, Austria).

## Results

**Fig. 1:**
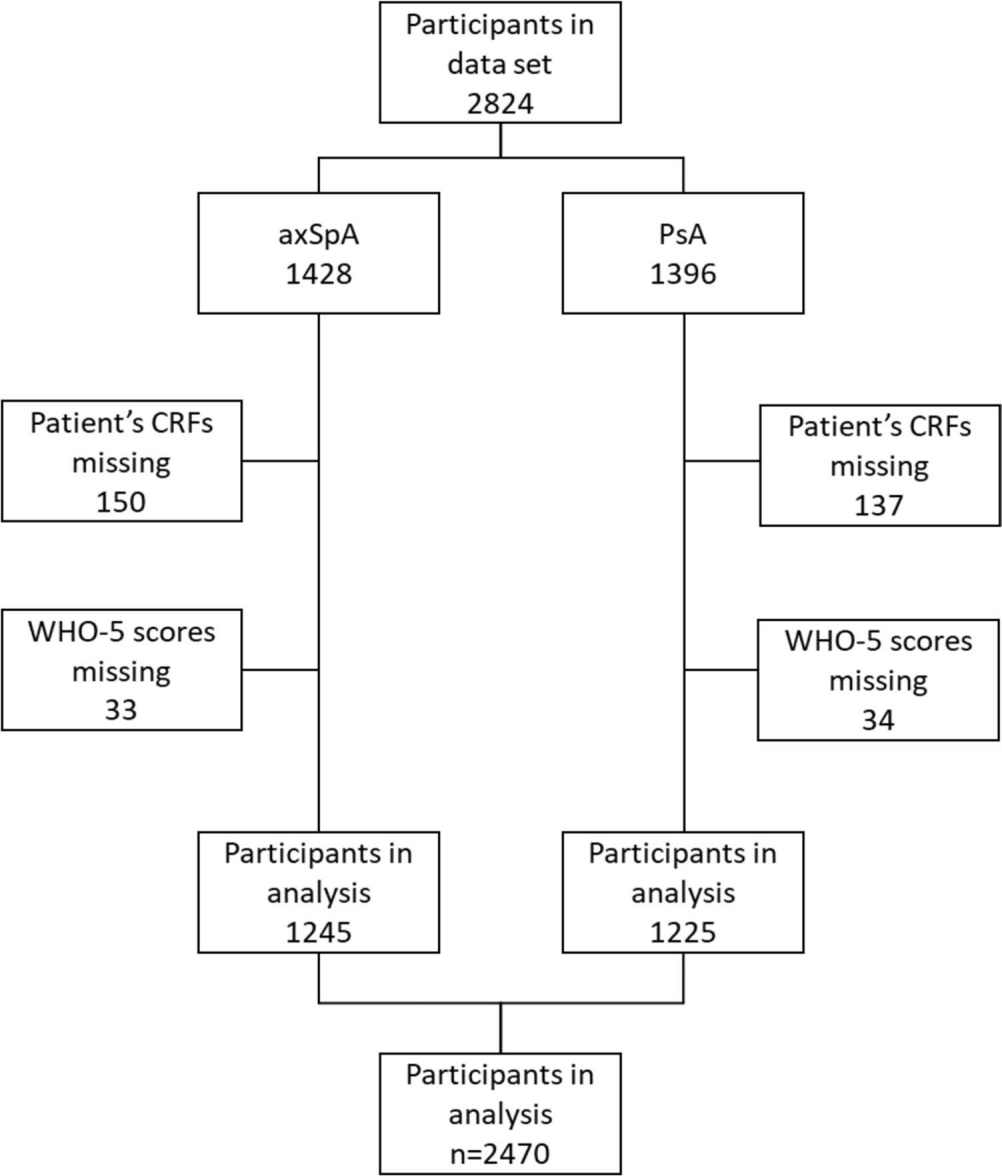
Patient selection chart

A total of 2470 patients were included in the analysis, 1245 with axSpA and 1225 with PsA. Patients were excluded if they had not submitted a patient’s questionnaire or if the WHO-5 score was missing or incomplete (see Fig. 1 for a detailed breakdown of patient selection).

### Comparison of sociodemographic and clinical characteristics of axSpA and PsA patients

As demonstrated in Table 1, PsA patients tended to be older and more often female than axSpA patients. A higher proportion were obese or had three or more comorbidities. Physician- and patient-reported disease activity was similar in both diagnoses. The distribution of WHO-5 scores was also similar across patients with axSpA and PsA diagnoses. Among PsA patients, 8.2% had severe and 20.9% had moderate depressive symptoms compared to 8.2% and 21.5%, respectively, among axSpA patients. See Table 1 for patient characteristics stratified by diagnosis.

**Table 1:** Characteristics of patients by diagnosis

	**axSpA**	**PsA**	**Total**
n	1245	1225	2470
Age, years, mean (SD)	44.5 ± 13	52.1 ± 12.3	48.3 ± 13.2
Sex, female, n (%)	561 (45)	719 (59)	1280 (52)
Obesity (BMI ≥ 30), n (%)	316 (26)	458 (38)	774 (32)
Naive to biologic therapy, n (%)	636 (61)	594 (57)	1230 (59)
Comorbidities, 3 or more, n (%)	241 (19)	382 (31)	623 (25)
Physician global disease activity, NRS 0–10, mean (SD)	5.4 ± 1.9	5.1 ± 1.9	5.3 ± 1.9
Patient global disease activity, NRS 0–10, mean (SD)	5.8 ± 2.4	5.7 ± 2.4	5.8 ± 2.4
WHO-5 score, mean (SD)	44 ± 21.4	45.8 ± 22.8	44.9 ± 22.1
WHO-5 score: Severe depressive symptoms, n (%)	102 (8)	101 (8)	203 (8)
WHO-5 score: Moderate depressive symptoms, n (%)	268 (22)	256 (21)	524 (21)
WHO-5 score: No or mild depressive symptoms, n (%)	875 (70)	868 (71)	1743 (71)

*NRS* Numerical rating scale, *WHO-5* WHO-5 Well-Being Index

### Comparison of characteristics by category of depressive symptoms in axSpA patients

Characteristics of axSpA patients, stratified by the three WHO-5 categories, are shown in Table 2. Patients with moderate as well as those with severe depressive symptoms were more often female than those with no or mild depressive symptoms, they were more often obese and fewer of them were currently employed. Patients with moderate or severe depressive symptoms had a slightly shorter disease duration, were less often naive to biologic therapy at the time of enrolment and a higher proportion of these patients were taking systemic corticosteroids and both non-opioid and opioid pain medication. They had a higher number of comorbidities and more often a depression as a comorbidity. With higher levels of depressive symptoms, all measures of disease activity and functional impairment, fatigue, pain and sleep disturbance worsened. Patients with moderate or severe depressive symptoms were more often current smokers, had a lower level of education and a lower proportion of them were engaging in sports for at least 1 h/week.

**Table 2:** Characteristics of axSpA patients by depressive symptoms

Parameter	No or mild depressive symptoms *N* = 875	Moderate depressive symptoms *N* = 268	Severe depressive symptoms *N* = 102	Total *N* = 1245
Age, years, mean (SD)	44.5 ± 13.2	43.9 ± 12.7	45.7 ± 11.8	44.5 ± 13
Sex, female, n (%)	384 (44)	128 (48)	49 (48)	561 (45)
Disease duration, years, mean (SD)	7.1 ± 9.3	6.5 ± 8.7	6.4 ± 8.1	6.9 ± 9.1
School, ≥ 10 years, n (%)	723 (84)	203 (78)	71 (70)	997 (81)
Currently employed, n (%)	688 (82)	184 (75)	71 (76)	943 (80)
Current smoking, n (%)	318 (37)	116 (44)	47 (48)	481 (40)
Engaging in sports, ≥ 1 h/week, n (%)	500 (57)	122 (46)	30 (29)	652 (53)
Obesity (BMI ≥ 30), n (%)	207 (24)	80 (30)	29 (28)	316 (26)
Comorbidities, 3 or more, n (%)	151 (17)	59 (22)	31 (30)	241 (19)
Depression as known comorbidity, n (%)	42 (5)	27 (10)	18 (18)	87 (7)
Of those, depression treated, n (%)	23 (55)	17 (63)	13 (72)	53 (61)
Fibromyalgia as comorbidity, n (%)	17 (2)	7 (3)	6 (6)	30 (2)
Naive to biologic therapy, n (%)	462 (63)	128 (56)	46 (49)	636 (61)
Systemic glucocorticoids, n (%)	142 (16)	57 (22)	21 (21)	220 (18)
Non-opioid analgesics, n (%)	84 (10)	39 (15)	18 (19)	141 (12)
Opioids, n (%)	39 (5)	21 (8)	11 (11)	71 (6)
HLA-B27 positive, n (%)	626 (76)	186 (74)	67 (68)	879 (75)
ASAS criteria fulfilled, n (%)	685 (78)	214 (80)	78 (76)	977 (78)
CRP ≥ 5 mg/l, n (%)	387 (52)	125 (55)	57 (63)	569 (54)
Arthritis, joint count (0–44), mean (SD)	0.9 ± 2.4	1.7 ± 4.1	0.6 ± 1.7	1.1 ± 2.8
Enthesitis, number of sites (0–16), mean (SD)	0.4 ± 1.3	0.6 ± 1.6	0.5 ± 1.4	0.5 ± 1.4
ASDAS (CRP), mean (SD)	2.6 ± 1	3.3 ± 0.9	3.7 ± 0.9	2.9 ± 1
Physician global disease activity, NRS 0–10, mean (SD)	5.2 ± 2	5.9 ± 1.6	6.2 ± 1.7	5.4 ± 1.9
BASDAI, mean (SD)	4.1 ± 1.8	5.8 ± 1.6	6.7 ± 1.5	4.7 ± 2
BASFI, mean (SD)	3.2 ± 2.2	5 ± 2.2	6.2 ± 2.1	3.8 ± 2.4
ASAS-HI Score (0–17), mean (SD)	5.7 ± 3.2	9.2 ± 3.1	11.2 ± 2.9	6.9 ± 3.7
ASAS-HI: good/very good (0–5), n (%)	404 (47)	29 (11)	3 (3)	436 (36)
ASAS-HI: moderate (6–12), n (%)	438 (51)	167 (65)	57 (57)	662 (54)
ASAS-HI: poor/very poor (> = 12), n (%)	22 (3)	61 (24)	40 (40)	123 (10)
Patient global disease activity, NRS 0–10, mean (SD)	5.2 ± 2.3	7 ± 1.8	7.6 ± 2	5.8 ± 2.4
Patient: Pain, NRS 0–10, mean (SD)	5.1 ± 2.3	6.8 ± 1.9	7.5 ± 1.8	5.6 ± 2.3
Patient: fatigue, NRS 0–10 mean (SD)	4.8 ± 2.3	6.9 ± 1.8	7.5 ± 1.8	5.5 ± 2
Patient: Sleep disturbance, NRS 0–10, mean (SD)	4.7 ± 2.9	6.9 ± 2.4	7.7 ± 2.4	5.4 ± 3
Patient: Current work ability, NRS 0–10 (higher is better), mean (SD)	5.6 ± 2.3	3.8 ± 2.5	3.1 ± 2.6	5.1 ± 2.6

*ASAS criteria* ASAS Criteria for Axial Spondyloarthritis, *ASAS-HI* Assessment of SpondyloArthritis International Society Health Index, *ASDAS (CRP)* Ankylosing Spondylitis Disease Activity Score c-reactive protein, *axSpA* Axial spondyloarthritis, *BASDAI* Bath Ankylosing Spondylitis Disease Activity Index, *BASFI* Bath Ankylosing Spondylitis Functional Index, *CRP* C-reactive protein, *HLA-B27* Human leukocyte antigen B27, *NRS* Numerical rating scale

### Comparison of characteristics by category of depressive symptoms in PsA patients

PsA patients in all three groups compared were of similar age (Table 3). Patients with moderate or severe depressive symptoms were more often female, fewer of them had at least 10 years of school education or were currently employed. Among patients with severe depressive symptoms, the percentages of obese patients and those taking pain medications were substantially higher than in the two other groups, and this was even more pronounced than in axSpA. Unlike axSpA patients, PsA patients with severe depressive symptoms had the longest disease duration. In patients with moderate or severe depressive symptoms, more had at least three comorbidities. The proportion of patients with documented depression or fibromyalgia was higher in the groups of patients with moderate or severe depressive symptoms. Just as in axSpA patients, measures of disease activity and functional impairment, fatigue, pain and sleep disturbance were all worse in those with more severe depressive symptoms compared to patients with no or mild depressive symptoms.

**Table 3:** Characteristics of PsA patients by depressive symptoms

Parameter	No or mild depressive symptoms *N* = 868	Moderate depressive symptoms *N* = 256	Severe depressive symptoms *N* = 101	Total *N* = 1225
Age, years, mean (SD)	52.3 ± 12.8	51.3 ± 10.9	52.8 ± 11.1	52.1 ± 12.3
Sex, female, n (%)	489 (56)	158 (62)	72 (71)	719 (59)
Disease duration, years, mean (SD)	6 ± 7.1	6.7 ± 8.3	7.8 ± 8.1	6.3 ± 7.4
School, ≥ 10 years, n (%)	680 (80)	192 (76)	75 (75)	947 (79)
Currently employed, n (%)	555 (67)	155 (65)	52 (54)	762 (66)
Current smoking, n (%)	230 (28)	82 (33)	37 (37)	349 (30)
Engaging in sports, ≥ 1 h/week, n (%)	386 (46)	87 (35)	29 (29)	502 (42)
Obesity (BMI ≥ 30), n (%)	304 (36)	98 (39)	56 (55)	458 (38)
Comorbidities, 3 or more, n (%)	247 (29)	89 (35)	46 (46)	382 (31)
Depression as known comorbidity, n (%)	64 (7)	60 (23)	25 (25)	149 (12)
Of those, depression treated, n (%)	44 (69)	39 (65)	19 (76)	102 (68)
Fibromyalgia as comorbidity, n (%)	30 (4)	21 (8)	11 (11)	62 (5)
Naive to biologic therapy, n (%)	442 (60)	107 (50)	45 (50)	594 (57)
Systemic glucocorticoids, n (%)	255 (30)	92 (36)	46 (46)	393 (32)
Non-opioid analgesics, n (%)	92 (12)	36 (16)	33 (34)	161 (15)
Opioids, n (%)	31 (4)	21 (9)	15 (16)	67 (6)
CRP ≥ 5 mg/l, n (%)	314 (41)	104 (44)	41 (45)	459 (42)
Tender joint count (0–68), mean (SD)	6.4 ± 7.7	7.2 ± 7.2	11.1 ± 10.7	6.9 ± 8.0
Swollen joint count (0–66), mean (SD)	3.0 ± 4.1	3.5 ± 4.7	4.7 ± 6.2	3.3 ± 4.4
Enthesitis, number of sites (0–16), mean (SD)	2.9 ± 2.5	3.6 ± 2.1	3.8 ± 3.2	3.2 ± 2.5
Nail psoriasis, n (%)	344 (40)	103 (40)	50 (50)	497 (41)
Body surface area, mean (SD)	8.3 ± 14.6	8.9 ± 16.2	10.2 ± 17.9	8.6 ± 15.2
DAPSA, mean (SD)	20.6 ± 12.5	24.8 ± 12.7	32.6 ± 15.9	22.5 ± 13.3
Physician global disease activity, NRS 0–10, mean (SD)	4.9 ± 1.8	5.5 ± 1.8	6.3 ± 1.6	5.1 ± 1.9
Patient global disease activity, NRS 0–10, mean (SD)	5.2 ± 2.3	6.6 ± 2.1	8 ± 2	5.7 ± 2.4
Patient: DLQI, mean (SD)	4.9 ± 5.5	7.2 ± 6.9	9.2 ± 8.2	5.8 ± 6.2
Patient: HAQ, mean (SD)	0.8 ± 0.6	1.2 ± 0.6	1.6 ± 0.6	0.9 ± 0.7
Patient: Fatigue, NRS 0–10, mean (SD)	4.4 ± 2.2	6.8 ± 2.0	8.1 ± 1.7	5.2 ± 2.7
Patient: Pain, NRS 0–10, mean (SD)	5 ± 2.3	6.4 ± 2.1	7.8 ± 1.7	5.5 ± 2.4
Patient: Sleep disturbance, NRS 0–10, mean (SD)	4.2 ± 2.9	6.3 ± 2.7	7.2 ± 2.7	4.9 ± 3.1
Patient: Current work ability, NRS 0–10 (higher is better), mean (SD)	5.6 ± 2.8	3.7 ± 2.7	3.5 ± 2.9	5 ± 2.9

*CRP* C-reactive protein, *DAPSA* Disease Activity in Psoriatic Arthritis, *DLQI* Dermatology Life Quality Index, *HAQ* Health Assessment Questionnaire, *NRS* Numerical rating scale

### Discrepancy between physician-reported and patient-reported disease activity

**Fig. 2:**
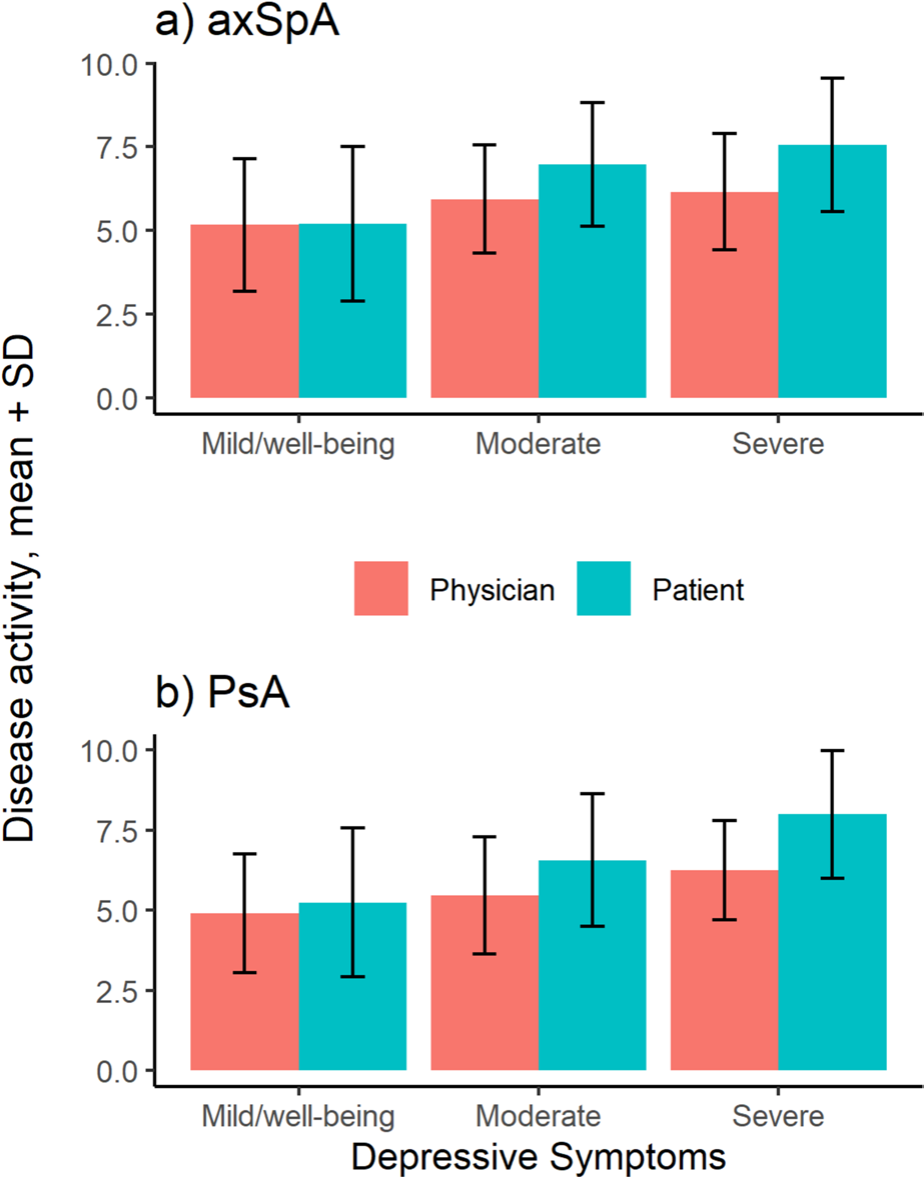
Physician-rated vs. patient-rated global disease activity by category of depressive symptoms **a**) axSpA; **b**) PsA. axSpA: Axial spondyloarthritis; PsA Psoriatic arthritis

In both axSpA and PsA patients, the mean values of physician and patient-reported global disease activity were remarkably similar in the group of patients with no or mild depressive symptoms (Fig. 2a  and  b). However, patients with moderate depressive symptoms rated themselves worse than their rheumatologist and in patients with severe depressive symptoms, that discrepancy was even greater.

### Factors associated with the presence of depressive symptoms

**Fig. 3:**
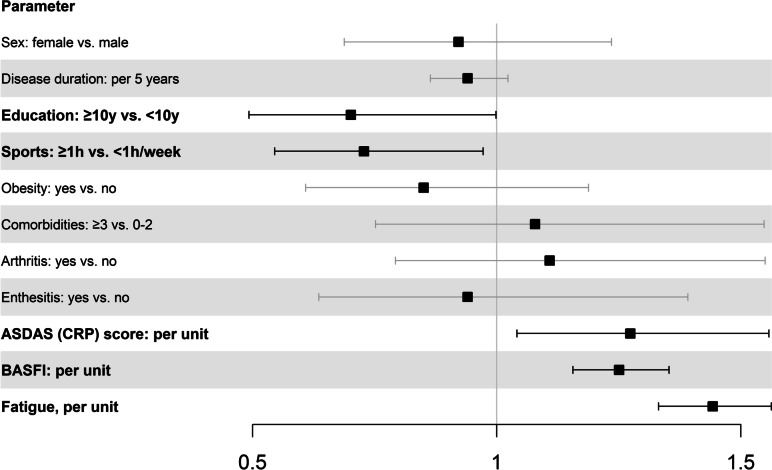
Adjusted OR (± 95% CI) of factors associated with the presence of symptoms suggestive of depression (WHO-5 score of ≤ 28) in axSpA patients: results from multivariable logistic regression. ASDAS (CRP): Ankylosing Spondylitis Disease Activity Score; BASFI: Bath Ankylosing Spondylitis Functional Index c-reactive protein

AxSpA patients with 10 or more years of education (OR = 0.70, 95% CI = 0.49–1.00) and patients who engaged in sports for at least 1 h a week (OR = 0.73, 95% CI = 0.54–0.97) were less likely to experience depressive symptoms. Patients with a higher disease activity as measured by ASDAS (CRP) (OR = 1.27, 95% CI = 1.04–1.56) and a higher functional impairment as measured by BASFI (OR = 1.25, 95% CI = 1.15–1.35) were more likely to experience depressive symptoms. Fatigue was most strongly associated with depressive symptoms (OR = 1.44, 95% CI = 1.33–1.56), see Fig. 3, Suppl. Table S1.

**Fig. 4:**
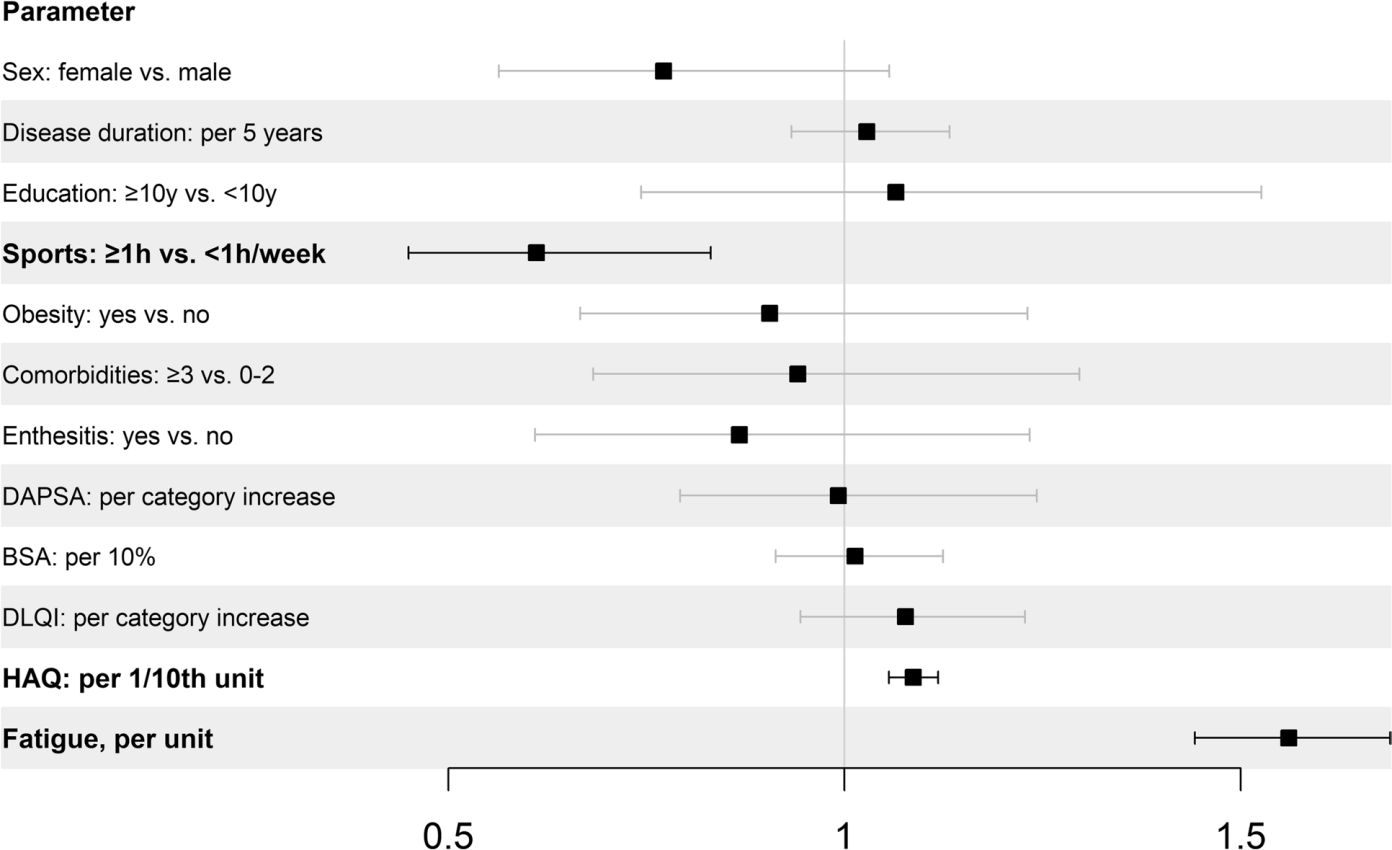
Adjusted OR (± 95%CI) of factors associated with the presence of symptoms suggestive of depression (WHO-5 score of ≤ 28) in PsA patients: results from multivariable logistic regression. BSA: Body surface area; DAPSA: Disease Activity in Psoriatic Arthritis; DLQI: Dermatology Life Quality Index; HAQ: Health Assessment Questionnaire

PsA patients who engaged in sports for at least 1 h a week (OR = 0.61, 95% CI = 0.45–0.83) were less likely to experience depressive symptoms. Patients with higher functional impairment as measured by HAQ (OR = 1.08, 95% CI = 1.06–1.12) were more likely to experience depressive symptoms. As in axSpA patients, the strongest association was with fatigue (OR = 1.56, 95% CI = 1.44–1.69), see Fig. 4, Suppl. Table S2.

## Discussion

Depressive symptoms are common in axSpA as well as in PsA with a prevalence of almost 30% in our cohort. The multivariate analysis showed that depressive symptoms are associated with fatigue, not engaging in sports and increased functional impairment in both diagnoses. In axSpA patients, fewer years in education and higher disease activity measured by ASDAS (CRP) are also associated with depressive symptoms.

The WHO-5 has been shown to be a suitable screening tool for depression [16, 22] and has been validated in a number of populations and settings [17, 23–27]. A study comparing the WHO-5, Patient Health Questionnaire 9 (PHQ-9) and Hospital Anxiety and Depression Scale (HADS) for the purpose of screening for depressive disorders, using the Structured Clinical Interview for DSM-IV as the criterion standard, found all three to perform well [16].

A recent systematic review of 39 studies, measuring the prevalence of depression or depressive symptoms in axSpA patients, found a large heterogeneity with prevalence estimates ranging from 10 to 64%. This is due, at least in part, to the use of different instruments to measure depression and to the use of different cut off values [6]. A previous analysis by our group [5], also using the WHO-5, found a prevalence of moderate to severe depressive symptoms of 31% in axSpA patients in Germany, using claims data that were linked to a cross-sectional survey. This is very similar to the prevalence we found in our cohort of patients from a German disease register.

Similarly heterogeneous are the studies on the prevalence of depression in PsA patients, summarised by Zusman et al. [8]. The authors found 18 studies reporting on the prevalence of depression, with prevalences ranging from 9 to 70%. The pooled prevalence was 17% (95% CI 13 – 21%). Depression was assessed using the PHQ-9, the HADS with different cut-offs or a documented ICD-9 or ICD-10 code. The prevalence found in our analysis fits well into the described range.

In the aforementioned analysis of factors associated with an outcome of moderate to severe depressive symptoms (≤ 28 points in the WHO-5) in axSpA patients [5], the authors identified a higher disease activity (BASDAI), functional impairment (BASFI), a lower income, self-reported stress and lack of exercise, and younger age as factors associated with moderate-to-severe depressive symptoms. A systematic review identified eight studies that were examining the association between depression and disease activity or functional impairment. All studies reported significantly poorer BASDAI and BASFI values for patients with depression compared to those without [6]. Another analysis, using longitudinal data and general estimating equation models to examine associations between sociodemographic and disease-specific factors and well-being (measured with the Bath Ankylosing Spondylitis Patient Global Score), found reduced well-being to be associated with pain, fatigue and physical functioning but not sociodemographic factors like age, sex or educational level [28].

In our analysis, not engaging in sports, disease activity (ASDAS (CRP)) and impaired functional capacity as well as fatigue were associated with depressive symptoms in axSpA patients, while education was a protective factor. We decided to use the composite score ASDAS (CRP) as disease activity measure, because it has emerged as the most appropriate instrument for disease activity [29].

Relatively few analyses of factors associated with depressive symptoms in PsA patients were found by us. One [30] showed abnormal physical function (Health Assessment Questionnaire Disability Index (HAQ-DI) ≥ 0.5) and elevated Interleukin-6 to be associated with depressive symptoms, another [31] identified DAPSA and HAQ-DI as being associated with depressive symptoms. In another study [32], patients who were employed were less likely to experience depressive symptoms while those who exhibited higher levels of fatigue were more likely to experience depressive symptoms. We identified not engaging in sports, impaired physical function and fatigue as factors associated with depressive symptoms. DAPSA, a recommended composite score to measure disease activity [20], was not associated with depressive symptoms.

Disease activity in axSpA, as measured by ASDAS (CRP), was associated with depressive symptoms. In contrast, there was no correlation between depressive symptoms and disease activity by DAPSA score in PsA patients. ASDAS (CRP) relies on PROMs (pain, morning stiffness and patient global disease activity) and CRP while DAPSA relies on tender and swollen joint count, CRP, and PROMs (patient global disease activity and pain). Hence, these composite scores measure disease activity very differently and the influence of PROMs on the total score result is much higher in the case of ASDAS (CRP). There is data suggesting that PROMs like fatigue and pain are closely related to mental health [33, 34] and therefore composite scores that rely mainly on PROMs are more influenced by mental health. An analysis from the Danish DANBIO Registry indicates that there may be a risk of overtreatment in rheumatoid arthritis patients with psychological problems or central sensitization where the disease activity calculation is based mainly on PROMs [35]. Another analysis from Portugal compared patients in remission to patients where only an insufficient patient global assessment prevented the achievement of remission [36]. The authors conclude that a modified treat to target approach, distinguishing between (objective) disease activity and the impact of disease on patients as a separate target might be beneficial and avoid unnecessary therapy escalations.

The association of fatigue with depressive symptoms is expected, as fatigue is an important characteristic of depression. Fatigue is a very common symptom in axSpA [37] and PsA [38]. As the first question of the BASDAI covers fatigue it is not surprising that the total BASDAI score is also influenced by depressive symptoms as shown previously [5].

The impact of depression or other mental health problems such as anxiety on the effectiveness of antirheumatic treatments has not been studied extensively yet. In a study from the Toronto PsA clinic, the probability of reaching minimal disease activity was reduced in the presence of depressive symptoms [11]. Similar results were found by Michelsen et al. for Rheumatoid arthritis and PsA [9]. In an analysis from the British Society for Rheumatology Biologics Register in Ankylosing Spondylitis (BSRBR-AS), the authors found a higher number of comorbidities and poor mental health to be negatively associated with treatment response, as were sociodemographic factors like fewer years in education and not working full-time [10]. In a follow-up analysis the authors found that a reduction of depressive symptoms from moderate/severe to ‘less than mild’ (0–7 points as measured by HADS) would improve treatment response by approximately 2 BASDAI units and reduce treatment discontinuation by a third [39]. Another analysis from BSRBR-AS showed symptoms of anxiety and depression to be independently associated with poorer treatment outcomes, both continuous disease parameters and binary response criteria.

Interestingly, we found an association between depressive symptoms and a discordance between physician and patient rated global disease activity. In patients with no or mild depressive symptoms, the mean values of patient and physician global disease activity were concordant. However, patients with moderate depressive symptoms rated their disease activity higher, and in patients with severe depressive symptoms, this discordance was even more pronounced. Discordance between physician and patient scores of global disease activity has been described before. In axSpA patients, Desthieux et al. [40] found the proportion of patients with a discordant disease activity assessment (defined as a difference of at least three points) to be between 26 and 29% from baseline to three years after baseline. Discordance was associated with spine pain and fatigue.

It has been shown that the discrepancy between patient- and physician- rated symptom severity is associated with depression [41]. Similar results were also found in psoriasis, with greater degree of discordance in patients with mental health disorders [42].

This discordance has implications for shared decision making in chronic conditions, which relies on similar assessment of disease states by patient and physician. Disagreement on disease activity and treatment success may lead to reduced patient satisfaction with treatment decisions and thus reduced adherence and treatment response. Physicians need to be aware of both comorbid depressive symptoms and the associated risk of discordant assessment of disease activity.

Another interesting finding in our analysis is the increasing intake of analgesics including opioids in axSpA and PsA patients associated with depressive symptoms. This goes along with higher levels of pain.

A limitation of the present analysis is the instrument to measure depressive symptoms, the WHO-5. This instrument was chosen because it is a validated screening tool for depression. It consists of only five questions and is therefore feasible in large longitudinal cohort studies with multiple research questions. The feasibility is evident from the very low number of missing values for the WHO-5 score. However, it is not a diagnostic instrument, therefore, our data needs to be interpreted carefully. However, we are confident that our results regarding depressive symptoms are robust and indicate a clinically relevant problem. Another limitation is that patients are included into our cohort at the start of a new systemic therapy, hence in a situation in which there is a need for treatment intensification. While it is a strength that this is the same for all patients, it can be expected that patients are at a low point and may exhibit more severe depressive symptoms than before or after inclusion. Another important limitation is the cross-sectional design of this analysis, therefore no causal relationships can be analysed.

## Conclusion

In our study of patients starting a new systemic therapy, depressive symptoms are common in axSpA and PsA. Depressive symptoms are associated with fatigue, not engaging in sports, and limited physical function. We conclude that mental health needs to be taken into account when assessing a patient’s situation in a more holistic way. A multidisciplinary treatment approach including psychotherapeutic interventions may help to optimize the management of disease in axSpA and PsA patients. A better understanding of the influence of depressive symptoms on the effectiveness of the antirheumatic treatment is an important future research task.

## Supplementary Information

**Additional file 1: Table S1.** Adjusted OR (±95% CI) of parameters associated with the presence of symptoms suggestive of depression (WHO-5 score of ≤28) in axSpA patients: results from multivariable logistic regression. **Table S2.** Adjusted OR (±95% CI) of parameters associated with the presence of symptoms suggestive of depression (WHO-5 score of ≤28) in PsA patients: results from multivariable logistic regression.

## Data Availability

The datasets used and/or analysed during the current study are available from the corresponding author on reasonable request.

## Abbreviations

ASAS criteria: ASAS Criteria for Axial Spondyloarthritis
ASAS-HI: Assessment of SpondyloArthritis International Society Health Index
ASDAS (CRP): Ankylosing Spondylitis Disease Activity Score
axSpA: Axial spondyloarthritis
BASDAI: Bath Ankylosing Spondylitis Disease Activity Index
BASFI: Bath Ankylosing Spondylitis Functional Index
bDMARD: Biologic disease modifying anti-rheumatic drug
BSA: Body surface area
BSRBR-AS: British Society for Rheumatology Biologics Register in Ankylosing Spondylitis
CRF: Case report form
CRP: C-reactive protein
DAPSA: Disease Activity in Psoriatic Arthritis
DLQI: Dermatology Life Quality Index
HADS: Hospital Anxiety and Depression Scale
HAQ: Health Assessment Questionnaire
HAQ-DI: Health Assessment Questionnaire Disability Index
HLA-B27: Human leukocyte antigen B27
PHQ-9: Patient Health Questionnaire 9
PROM: Patient reported outcome measure
PsA: Psoriatic arthritis
tsDMARD: Targeted synthetic disease modifying anti-rheumatic drug
WHO-5: WHO-5 Well-Being Index
