# Correction: Depressive symptoms are associated with fatigue, poorer functional status and less engagement in sports in axSpA and PsA: an analysis from the RABBIT-SpA cohort

**DOI:** 10.1186/s13075-024-03411-9

**Published:** 2024-10-11

**Authors:** Andreas Reich, Anja Weiß, Lisa Lindner, Xenofon Baraliakos, Denis Poddubnyy, Silke Zinke, Carsten Stille, Anja Strangfeld, Anne C. Regierer

**Affiliations:** 1 German Rheumatism Research Center (DRFZ Berlin), Epidemiology and Health Services Research, Berlin, Germany; 2 Ruhr University Bochum, Bochum, Germany; 3 Rheumazentrum Ruhrgebiet, Herne, Germany; 4 Charité – Universitätsmedizin Berlin, Department of Gastroenterology, Infectiology and Rheumatology, Berlin, Germany; 5 Berlin, Germany; 6 Hannover, Germany; 7 Charité – Universitätsmedizin Berlin, Berlin, Germany

**Correction: Arthritis Res Ther 25, 136 (2023)**

10.1186/s13075-023-03127-2.

Following publication of the original article [1], the authors reported an error under the Abstract, Result sub-section.

The sentence currently reads:

In both diagnoses, the proportion of patients with moderate depressive symptoms was 8% and 21% with severe symptoms.

The sentence should read:

In both diagnoses, 21% of patients exhibited moderate and 8% severe depressive symptoms.

## References

[1] Reich A, Weiß A, Lindner L, et al. Depressive symptoms are associated with fatigue, poorer functional status and less engagement in sports in axSpA and PsA: an analysis from the RABBIT-SpA cohort. Arthritis Res Ther. 2023;25(1):136. 10.1186/s13075-023-03127-2. 37533077 10.1186/s13075-023-03127-2 PMC10394807

