# Supplementary material for: Depressive symptoms are associated with fatigue, poorer functional status and less engagement in sports in axSpA and PsA: an analysis from the RABBIT-SpA cohort
Source: Arthritis Res Ther. 2023 Aug 2;25:136. doi: 10.1186/s13075-023-03127-2 (PMC10394807; doi:10.1186/s13075-023-03127-2)
Supplement: Supplementary file 1 — Additional file 1: Table S1. Adjusted OR (±95% CI) of parameters associated with the presence of symptoms suggestive of depression (WHO-5 score of ≤28) in axSpA patients: results from multivariable logistic regression. Table S2. Adjusted OR (±95% CI) of parameters associated with the presence of symptoms suggestive of depression (WHO-5 score of ≤28) in PsA patients: results from multivariable logistic regression. [file 13075_2023_3127_MOESM1_ESM.docx]

Table S1: Adjusted OR (±95% CI) of parameters associated with the presence of symptoms suggestive of depression (WHO-5 score of ≤28) in axSpA patients: results from multivariable logistic regression

| **Parameter** | **Odds Ratio** | **95% Confidence interval** | | **P-value** |
| --- | --- | --- | --- | --- |
| Sex: female vs. male | 0.92160 | 0.68771 | 1.23504 | 0.5846 |
| Disease duration: per 5 years | 0.94050 | 0.86439 | 1.02332 | 0.1543 |
| Education: >=10y vs. <10y | 0.70140 | 0.49259 | 0.99871 | 0.0492 |
| Sports: >=1h vs. <1h/week | 0.72822 | 0.54537 | 0.97238 | 0.0316 |
| Obesity: yes vs. no | 0.85026 | 0.60853 | 1.18803 | 0.3419 |
| Comorbidities: >=3 vs. 0-2 | 1.07857 | 0.75171 | 1.54757 | 0.6814 |
| Arthritis: yes vs. no | 1.10831 | 0.79248 | 1.55001 | 0.5479 |
| Enthesitis: yes vs. no | 0.94048 | 0.63563 | 1.39154 | 0.7588 |
| ASDAS (CRP) score: per unit | 1.27369 | 1.04160 | 1.55750 | 0.0187 |
| BASFI: per unit | 1.25066 | 1.15615 | 1.35289 | <.0001 |
| Fatigue, per unit | 1.44243 | 1.33146 | 1.56264 | <.0001 |

Table S2: Adjusted OR (±95% CI) of parameters associated with the presence of symptoms suggestive of depression (WHO-5 score of ≤28) in PsA patients: results from multivariable logistic regression

| **Parameter** | **Odds Ratio** | **95% Confidence interval** | | **P-value** |
| --- | --- | --- | --- | --- |
| Sex: female vs. male | 0.77165 | 0.56366 | 1.05639 | 0.1057 |
| Disease duration: per 5 years | 1.02808 | 0.93329 | 1.13250 | 0.5747 |
| Education: >=10y vs. <10y | 1.06507 | 0.74322 | 1.52630 | 0.7313 |
| Sports: >=1h vs. <1h/week | 0.61130 | 0.44962 | 0.83111 | 0.0017 |
| Obesity: yes vs. no | 0.90574 | 0.66645 | 1.23095 | 0.5271 |
| Comorbidities: >=3 vs. 0-2 | 0.94093 | 0.68277 | 1.29671 | 0.7098 |
| Enthesitis: yes vs. no | 0.86716 | 0.60950 | 1.23374 | 0.4282 |
| DAPSA: per category increase | 0.99238 | 0.79240 | 1.24284 | 0.9469 |
| BSA: per 10% | 1.01325 | 0.91318 | 1.12429 | 0.8040 |
| DLQI: per category increase | 1.07698 | 0.94456 | 1.22797 | 0.2679 |
| HAQ: per 1/10th unit | 1.08666 | 1.05619 | 1.11800 | <.0001 |
| Fatigue, per unit | 1.56068 | 1.44226 | 1.68882 | <.0001 |
